# Testing rTMS-Induced Neuroplasticity: A Single Case Study of Focal Hand Dystonia

**DOI:** 10.1155/2018/6464896

**Published:** 2018-05-30

**Authors:** Sonia Betti, Andrea Spoto, Umberto Castiello, Luisa Sartori

**Affiliations:** ^1^Dipartimento di Psicologia Generale, Università di Padova, Padova, Italy; ^2^Centro Beniamino Segre, Accademia Nazionale dei Lincei, Roma, Italy; ^3^Centro di Neuroscienze Cognitive, Università di Padova, Padova, Italy

## Abstract

Focal hand dystonia in musicians is a neurological motor disorder in which aberrant plasticity is caused by excessive repetitive use. This work's purposes were to induce plasticity changes in a dystonic musician through five daily thirty-minute sessions of 1 Hz repetitive transcranial magnetic stimulation (rTMS) applied to the left M1 by using neuronavigated stimulation and to reliably measure the effect of these changes. To this aim, the relationship between neuroplasticity changes and motor recovery was investigated using fine-grained kinematic analysis. Our results suggest a statistically significant improvement in motor coordination both in a task resembling the dystonic-inducing symptoms and in a reach-to-grasp task. This single case study supports the safe and effective use of noninvasive brain stimulation in neurologic patients and highlights the importance of evaluating outcomes in measurable ways. This issue is a key aspect to focus on to classify the clinical expression of dystonia. These preliminary results promote the adoption of kinematic analysis as a valuable diagnostic tool.

## 1. Introduction

Dystonias are a group of disorders characterized by intermittent or sustained muscle contractions causing twisting and repetitive movements (for a review, see [1, 2]). The crucial catalyst behind dystonia is a multifactorial combination of excessive plasticity, intensive training, and failure of limiting plastic changes, as seen through noninvasive neurostimulation studies [3]. Once this abnormal plasticity process is brought under control, it could ultimately result in a clinical improvement [4].

Dystonia may be task-specific producing abnormal motor performance for only a specific task, such as in musician's dystonia (MD). MD affects isolated fingers that perform complex and repetitive motor tasks during actions associated with musical play, but can also lead to impaired adjacent finger flexion [2]. This overflow into adjacent muscles not specifically involved in the particular motor task is due to a loss of inhibition that manifests in the periphery with abnormally long muscle bursts [5]. In MD, abnormally prolonged muscle firing due to selective overtraining of an intended finger may prevent the ability to keep excitability within a useable range (i.e., homeostatic plasticity), a function which is specifically impaired in dystonia [6].

Although its underlying pathophysiology remains unclear, several studies in patients with MD have shown that repeated and prolonged hand use might result in abnormal activity in the cortical representation of the hand [7, 8]. In fact, important neural correlates of task-specific dystonia are the enlarged and partially overlapping fields revealed by brain imaging and transcranial magnetic stimulation (TMS) studies targeting the somatosensory and the motor cortices [8–14]. Whereas a typical homuncular organization reveals a distance of about 2.5 cm between the representations of the thumb and the little finger, these boundaries seem to be blurred for the dystonic fingers [10]. This lack of clearly defined somatosensory and motor cortical representations can lead to involuntary motor control [15]. The loss of control is particularly evident during fast passages, often leading to involuntary flexion or extension of one or more fingers [16]. In particular, stringed instrument players exhibit a use-dependent alteration in the cortical representational zones of the digits of the hand that engage in the dexterity-demanding task of fingering the strings [17]. While initial MD is only associated with impairment of highly practiced motor tasks, it can subsequently lead to severe deficits, eventually terminating a career for one percent of professional musicians [18].

Although prompt initiation of treatment could rescue some patients, dystonia is often misdiagnosed or neglected since the lack of objective diagnostic criteria and reliable biomarkers prohibits early diagnostic recognition [19]. So far, the extent of motor symptoms has mainly been estimated by means of visual inspection and rating procedures (e.g., [20]), without providing fine resolution (but see [21] for an example of kinematic analysis to assess a flautist performance). In addition, treatment responses are very patient-dependent. A precise quantification tool for objective and reliable diagnosis and for treatment evaluation is therefore needed to acquire highly precise data and to identify subtle differences in the symptomatology.

Given the sparse literature on this topic, there are no clinical practice guidelines on how to recover voluntary motor control. To date, the preferred treatment for dystonia is botulinum toxin injection, but it only transiently works in a minor fraction of patients and its application is limited by the spread of weakness to adjacent muscles, which causes further motor performance impairment [22].

Recently, motor training has been combined with neurostimulation methods in an attempt to normalize brain excitability and recover motor performance [23, 24]. Notably, since the effects of long-term treatment might differ from those of a single session [24], TMS is usually delivered in repeated daily sessions to prolong after effects. Therapeutic procedures with dystonic patients classically adopted daily sessions of low-frequency repetitive TMS (rTMS) over the primary motor cortex (M1; [5, 25–28]) or the premotor cortex (PM; [24, 29, 30]). Siebner and colleagues [25, 26] evaluated the effect of low-frequency (1 Hz) stimulation of M1 to increase inhibition in the motor areas of the cerebral cortex. Low-frequency rTMS set to 10% below the resting motor threshold of the target muscle restored intracortical inhibition. Treatment output on handwriting was quantified by means of a pressure-sensitive digitizing tablet.

Needless to say, the principle of the measurement must be based on the phenomenology of each patient. Motor assessment must be specifically related to the compromised movement (i.e., the particular exercise that most consistently induced the dystonic disorder), rather than to a more general skill (e.g., [21, 31]). As Pujol and colleagues (2000) convincingly demonstrated in an fMRI study, a tailored assessment of patients in the dystonia-inducing situation is necessary [8].

The aim of the present study was to test a multimethodological paradigm based on the combination of single-pulse transcranial magnetic stimulation (spTMS), low-frequency rTMS, and 3D motion analysis in a professional guitarist affected by MD. Single pulses of TMS were used to assess the excitability of synaptic connections within the motor cortex, providing indirect measures of changes produced by neural plasticity. In addition, TMS can also produce long-term changes in excitability if the TMS pulses are applied repetitively [27]. In both cases, changes in excitability were monitored by computing the amplitude of the motor-evoked potential (MEP) in response to a standard TMS pulse. In particular, resting motor threshold MEPs reflect the degree of corticospinal system activation and potentially help in diagnosing motor symptoms and in monitoring treatment progress (i.e., whether interventions are safe and effective in slowing symptoms). Fine-grained 3D movement analysis has been adopted to specifically evaluate the treatment both in terms of improved motor coordination and cortical plasticity. The acquisition of MEPs induced by spTMS to the left M1 and recorded from the contralateral second dorsal interosseous (SDI) muscle before and after five daily sessions of rTMS protocol allowed to measure the variations on the resting motor threshold to obtain a physiological index of *neural plasticity*. Moreover, we considered two behavioral measures of *performance plasticity*: (i) a repetitive sequence of fingers' movement (task 1) and (ii) a reach-to-grasp action (task 2). Since guitar arpeggios involve a rapid succession of fine and isolated finger movements, the finger flexion task was conceived as a realistic attempt to execute the affected flexion pattern. As concerns the grip task, it was specifically chosen to investigate the distinct contribution of the two separate reaching and grasping components [32] on performed movements: the timing dissociation between these two components may in fact give useful hints to the underlying pathological state [33, 34]. Notably, problems to grasp and manipulate objects are frequent in movement disorders and a methodological approach providing highly standardized measures of natural movements is needed [35]. The outputs of both tasks were compared at local and general levels: across daily sessions and throughout the intervention, to provide a consistent measure of plasticity trend.

## 2. Method

### 2.1. Participants

A 55-year-old male right-handed classical guitarist (M.C.) diagnosed as suffering from MD in his right hand was recruited at the Neuroscience of Movement (NEMO) Laboratory at the Department of General Psychology, University of Padua. He specifically presented a painless and exaggerated involuntary flexion pattern in his right middle finger's metacarpophalangeal joint, which occurred exclusively in the task-specific context of playing the musical instrument (i.e., plucking the strings). The loss of synergistic muscle control was also evident as a cocontraction of adjacent muscles. The onset of the movement disorder had been three years before this study and had forced him to interrupt his career as a professional musician and especially as a concert performer. He reported no dystonic movement patterns in other activities. There was no evidence of any other neurologic disorder and he was not under medication. An additional guitarist served as control subject in this study. He was right-handed with comparable experience (40 years of practice) and age (50 years).

No adverse effects were reported during the experiment. Informed consent was obtained after they were fully informed, according to the Declaration of Helsinki, about the study's nature. The experimental protocol was approved by the University of Padua Ethics Review Board.

### 2.2. General Procedure

**Figure 1:**
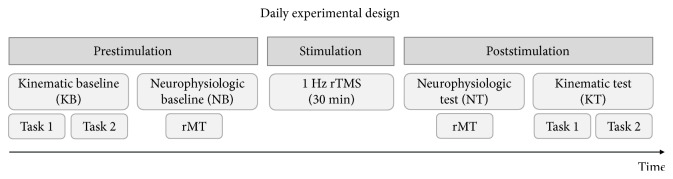
Daily experimental design for both MD patient and control participant. The graph represents the three daily phases of the experiment: behavioral and neurophysiological indexes were measured both before (prestimulation) and after (poststimulation) each stimulation session with low-frequency rTMS (1 Hz) on the left M1 (stimulation). The kinematic behavioral assessment (KB and KT) consisted of two tasks: finger abduction (task 1) and reach-to-grasp (task 2). In the neurophysiological assessment (NB and NT), the resting motor threshold was measured to assess corticospinal excitability variations. This protocol was repeated for five consecutive days.

A daily protocol (Figure 1) entailing two evaluation sessions (prestimulation) of kinematic and resting motor threshold (rMT) baselines, followed by low-frequency rTMS (stimulation) and kinematic and rMT tests (poststimulation), was repeated for five consecutive days and was designed as follows:

1. Prestimulation kinematic baseline (KB). A series of alternating finger flexion movements (i.e., the index, middle, and ring fingers, 15 movements per finger; task 1) with the palm upward and a sequence of 15 reach-to-grasp movements (task 2) were performed to test independent movements of the dystonic finger and motor coordination.
2. Prestimulation neurophysiologic baseline (NB). TMS-induced motor-evoked potentials (MEPs) were recorded from the right second dorsal interosseous (SDI) muscle to measure the rMT, thus assessing corticospinal excitability before intervention.
3. Stimulation. The participant underwent 30 minutes of rTMS (1 Hz) over the SDI muscle representation on the left primary motor cortex, delivered with intensity of 90% with respect to the rMT.
4. Poststimulation neurophysiologic test (NT). The same procedure adopted during the prestimulation NB session was implemented for comparison purposes. We performed a trend analysis to evaluate changes in motor cortex plasticity.
5. Poststimulation kinematic test (KT). The same procedure adopted during preintervention KB session was implemented for comparison purposes. We performed a day-by-day analysis and we compared the first and last day to evaluate both short- and long-term effects in motor coordination.

### 2.3. Kinematics Recording

**Figure 2:**
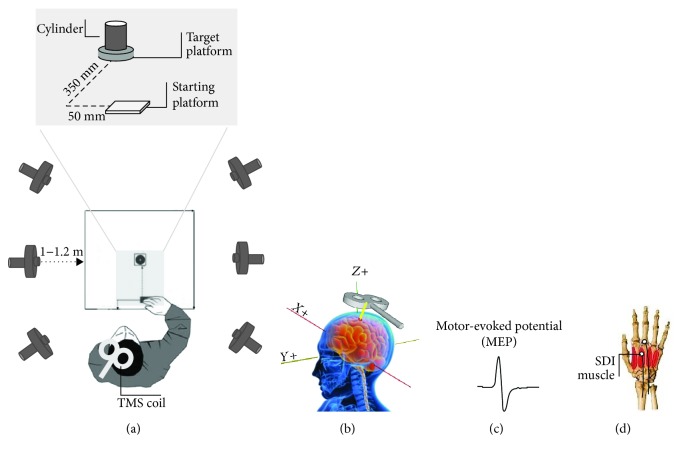
Experiment setup. A 3D optoelectronic SMART-D system was used to track the kinematics of the participant's right hand by means of six video cameras (a). TMS coil placement over the participant's left M1 hand area (b). Example of a TMS-evoked MEP (c). The targeted second dorsal interosseous (SDI) muscle (d).

A 3D optoelectronic SMART-D system (Bioengineering Technology and Systems, BTS) was used to track the kinematics of the participants' right hand. During the KB and KT phases, the participants were seated in a height-adjustable chair in front of a table (900 mm × 900 mm) with the right hand placed on a designated position on the table surface so as to guarantee the consistency of the start position across participants. Three semispherical infrared-reflective markers (5 mm diameter) were attached to the right hand on the tip of the index, middle, and ring fingers, and one was attached to the radial aspect of the wrist. Six digital video cameras with a frequency of 140 Hz were placed in a semicircle around the table (at 1–1.2 m away) to detect the markers (see Figure 2(a)). Before the experimental sessions, cameras position, roll angle, focus, zoom, brightness, and threshold were adjusted to optimize markers' tracking. Static and dynamic calibrations were then performed. For the static calibration, a three-axis frame of reference at known distance was placed on the center of the table. For the dynamic calibration, a three-marker wand was moved in all directions throughout the workspace of interest for approximately one minute. The spatial resolution of the recording system was 0.3 mm over the field of view. The standard deviation (SD) of the reconstruction error was below 0.2 mm for all the axes (*x*, *y*, and *z*).

During each daily session of the KB and KT phases, the participants took part in two tasks:

- Task 1: A series of 45 randomly alternating finger flexion movements of the index, middle, and ring fingers (15 movements per finger) were performed to test independent movements of the dystonic and adjacent fingers. The participants' right wrists were placed over a wooden cylinder (7.5 mm diameter; 11 cm high) with the palm of the hand facing upwards.
- Task 2: In the prehension task, a sequence of 15 reach-to-grasp movements was performed to test motor coordination. At the beginning of each trial, the hand was pronated with the palm resting on a starting platform (60 × 70 mm; 5 mm thick), which was shaped to allow for a comfortable and repeatable posture of all digits, that is, slightly flexed at the metacarpal and proximal interphalangeal joints. Then, the participants were asked to reach and grasp the cylinder located frontally with a whole hand grasp (WHG). The starting platform was attached 90 mm away from the edge of the table surface 50 mm away from the midsection. The cylinder was placed on a target platform, located at a distance of 350 mm from the starting platform, for consistent replacing (Figure 2(a)). An affixed colored dot on the cylinder was signaling the required thumb's contact point in order to perform stable and consistent grasps across the experiment. An auditory signal (300 Hz; 200 ms) was adopted as the “go” signal.

### 2.4. Transcranial Magnetic Stimulation and Electromyographic Recording

During both the MB and the MT phases, each participant was comfortably seated in an armchair with the right hand positioned on a pillow and the head kept stable by a neck pillow. The participant was asked to keep his muscles relaxed and to remain as still as possible during the delivery of the TMS pulses. While TMS pulses were delivered, he was asked to observe a white fixation cross on black background presented in the center of a monitor. TMS-induced MEPs were acquired from the participant's SDI muscle of the right hand (Figure 2(c)). EMG activity was recorded through pairs of surface Ag-AgCl surface electrodes (9 mm diameter) placed in a belly-tendon montage, with the active electrodes over the SDI muscle and the reference electrodes over the corresponding metacarpophalangeal joint (Figure 2(d)). The ground electrode was placed over the dorsal part of the right wrist. Electrodes were connected to an isolable portable ExG input box linked to the main EMG amplifier for signal transmission via a twin fiber optic cable (Professional BrainAmp ExG MR, Munich, Germany). Single-pulse TMS was administered using a 70 mm figure-of-eight coil connected to a Magstim Bistim^2^ stimulator (Magstim Co., Whitland, UK). Pulses were delivered to the hand region of the left M1. The coil was placed tangentially on the scalp, with the handle pointing laterally and caudally, so that the flow of induced electrical current in the brain travelled in a posterior-anterior direction [36, 37]. During the first session of the first day, the optimal cortical hotspot of the target muscle (OSP; i.e., the position at which larger and more stable MEPs are recorded from SDI with minimal stimulation intensity) was identified by delivering single TMS pulses at fixed intensity while moving the coil of 0.5 cm around the target area until the position was reached. To maintain an accurate and constant placement of the coil throughout the experimental sessions, it was kept over the OSP by a mechanical arm (Manfrotto, Italy) and its position and orientation were recorded and loaded into a neuronavigation system (SofTaxic Optic, EMS, Bologna, Italy; Figure 2(b)). Once the OSP was found, the individual resting motor threshold (rMT)—defined as the lowest stimulus intensity at which TMS is able to generate MEPs of at least 50 *μ*V in relaxed muscles in 5 out of 10 consecutive pulses [38]—was determined. rMT was then measured every day before and after the rTMS protocol to test possible variations of corticospinal excitability. Repetitive TMS pulses were applied using a Magstim Rapid^2^ stimulator (Magstim Co., Whitland, UK) with a figure-of-eight coil (70 mm outer diameter). Each rTMS session consisted of the application of off-line, low-frequency 1 Hz TMS for 30 min (1800 total pulses) at 90% of each participant's rMT. Both spTMS and rTMS were delivered on the side of the brain contralateral to the participant's dominant (and dystonia-affected) hand.

## 3. Data Analysis

### 3.1. Behavioral Measures

Following kinematic data collection, each trial was individually checked for correct marker identification and then run through a low-pass Butterworth filter with a 6 Hz cutoff. The SMART-D Tracker software package (Bioengineering Technology and Systems, BTS) was employed to reconstruct the 3D marker positions as a function of time. In task 1, the amplitude of maximum 3D distance between the dystonic fingertip and adjacent fingers (i.e., index and ring tips) was calculated as an index of abduction independence (AI). The amplitude of minimum distance between the dystonic finger and wrist was calculated as an index of abduction degree (AD) and compared to adjacent fingers' AD (see [21] for a similar approach). In task 2, we selected a set of standard measures universally reported in the literature for reach-to-grasp tasks, possibly enabling a productive comparison of results across participants (MD; control) and across experiments. We first computed movement onset (i.e., the first time point at which the wrist velocity crossed a 5 mm/sec threshold and remained above it for longer than 100 ms) and time of grip offset (i.e., the time at which the grip velocity dropped below a 5 mm/s threshold). Then, the following indexes were measured:

- Movement time (i.e., the time interval between onset and offset)
- Maximum grip aperture (MGA, the maximum distance reached by the 3D coordinates of the thumb and index finger)
- Time of maximum grip aperture (TMGA, the time at which the distance between the 3D coordinates of the thumb and index finger was maximum from movement onset)
- Time of maximum grip velocity (TMGV, the time at which the tangential velocity of the 3D coordinates of the thumb and index finger was maximum from movement onset)
- Time of maximum wrist height (TMWH, the time at which the 3D coordinates of the wrist were maximum from movement onset)
- Time of maximum wrist deceleration (TMWD, the time at which the deceleration of the 3D coordinates of the wrist was maximum from movement onset)
- Delay grasping (DG, the time interval between the onset of the wrist movement and the onset of fingers' opening)

### 3.2. Neurophysiological Measures

Motor threshold at rest before (rMT pre) and after (rMT post) rTMS 1 Hz stimulation was evaluated in both MD and control participants.

### 3.3. Statistical Analyses

**Figure 3:**
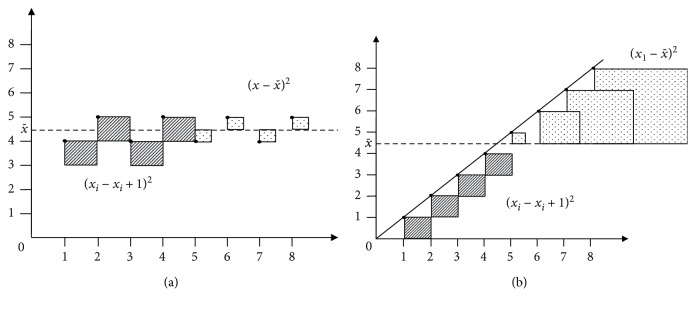
Graphical representation of the squared difference between (i) each data point and its subsequent value in the series (lined squares) and (ii) each data point and the average of the series (dotted squares) in the case of an oriented trend (a) and in the case of stationary data (b).

Behavioral data were analyzed using the R 3.3.9 statistical package [39]. More specifically, data were analyzed by means of an ad hoc function developed to implement the computation of the Young *C* test statistics [40]. This test, proposed by Young and Von Neumann, is used to evaluate the presence of a trend into a sequence of data collected on the same subject. It computes the probability that a sequence of data points follows a random, nonoriented distribution. If this probability is low, then the presence of some sort of either increasing or decreasing trend in the data can be argued. More specifically, the *C* test statistic is computed according to the following formula:

(1) $$\begin{array}{c} C=1-\frac{\sum_{i=1}^{N-1}{\left(x_{i}-x_{i+1}\right)}^{2}}{2\sum_{i=1}^{N-1}{\left(x_{i}-\bar{x}\right)}^{2}}, \end{array}$$

where *N* is the number of observations; *x*_*i*_ and $\bar{x}$ are the average values of the observations. The value of *C* tends to increase as an inverse function of the ratio between the squared difference of each data point to its subsequent and the squared difference of each point to the mean. The smaller the ratio, the higher the *C*, the higher the probability that the data do follow some sort of oriented trend (Figure 3).

Given these premises, data analysis was carried out on the two different tasks. More specifically, with respect to task 1, a comparison between the trend of values obtained during the first and the last day of training was carried out separately for the data collected before (pre) and after (post) the administration of the rTMS protocol. Moreover, an analysis of the overall trend along all the five days of training was conducted. This analysis was carried out separately for the pre- and post-rTMS phases and for the variables measured in task 2. A pointwise difference (delta) between the values obtained at the pre- and post-rTMS phases has been computed in order to highlight the presence of any particular daily pattern. As concerns motor threshold at rest, the presence of a significant trend before (rMT pre) and after (rMT post) rTMS 1 Hz stimulation was evaluated by means of the test *C* in both MD and control participants.

## 4. Results

### 4.1. Behavioral Plasticity

#### 4.1.1. Task 1: Finger Flexion Task

**Figure 4:**
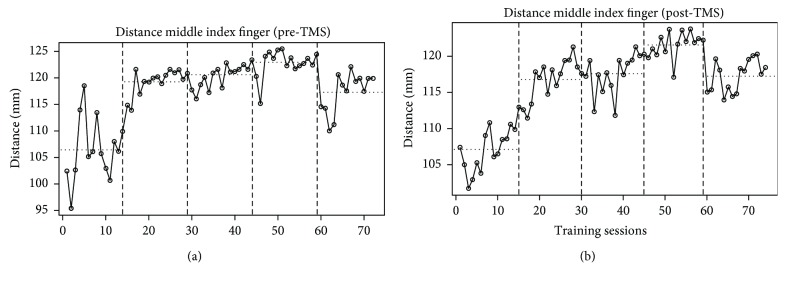
Graphical representation of the trend observed at the pre-rTMS (a) and post-rTMS (b) phases for the distance between the middle and index fingers when the former was flexed.

The main reference point of the analysis was the movement involving the middle finger. In the MD post phase, a significant increasing trend in the distance between the dystonia-affected finger and the index finger (AI) was observed on the first day of training (*C* = .66; *p* < .01). The evaluation of the overall trend showed that the measures collected throughout all the five days followed a significantly increasing trend (*C* = .87; *p* < .01). Reverse considerations can be referred to the pre-rTMS measures: a significantly increasing trend was not observed at the first day of training (*C* = .20; n.s.); however, the data collected the last day presented a significantly increasing trend (*C* = .54; *p* < .05); the overall trend was significantly increasing (*C* = .81; *p* < .01).  Figure 4 shows the increasing trend of this measure during the five days of training both at the pre-rTMS phase (a) and at the post-rTMS phase (b).

The same analysis conducted for the distance between the middle finger and the ring finger shows slightly different results. More specifically, while for the post measures the trend observed on the first day was significant (post: *C* = .62; *p* < .01), the measures collected in the pre phase did not show any trend (pre: *C* = .33; n.s.). Moreover, none of the trends were significant in the last days of training (day 5; post: *C* = .40; n.s.; pre: *C* = .06; n.s.). On the other hand, both the overall trends resulted significant (post: *C* = .65; *p* < .01; pre: *C* = .64; *p* < .01), showing an overall increase of the distance between the two fingers throughout the protocol. As concerns the control conditions (i.e., flexion of the adjacent fingers), no significantly increasing trend was observed in the distance between the index finger and the middle finger either at the first day of the training (pre: *C* = .30; n.s.; post: *C* = .37; n.s.) or at the last day (pre: *C* = .32; n.s.; post: *C* = .33; n.s.) when the index finger was flexed. Moreover, the overall analysis showed a significant trend in the pre-rTMS phase (*C* = .57; *p* < .01) while no effect was found in the post (*C* = .37; n.s.). The same results emerged from the analysis of the distance between the index finger and the ring finger when the former was flexed. More precisely no significant trend was observed neither at the first day of the training phases (pre: *C* = .28; n.s.; post: *C* = .01; n.s.) or at the last day of training (pre: *C* = .60; n.s.; post: *C* = .03; n.s.). The overall analysis showed a significant trend in the pre-TMS phase (*C* = .62; *p* < .01) while no effect was found in the post (*C* = .37; n.s.). Finally, when the ring finger was flexed only, some of the overall trends were significantly increasing, namely, the distance between the ring finger and the index finger in the pre-rTMS phase (*C* = .75; *p* < .01) and the distance between the ring finger and the middle finger in both phases (pre: *C* = .78; *p* < .01; post: *C* = .85; *p* < .01). No significant overall trend was observed for the distance between the ring finger and the index finger during the post-rTMS phase (*C* = .40; n.s.). In terms of the middle finger abduction degree, a significant decreasing trend was observed the first day of training at the post-TMS phase (*C* = .69; *p* < .01). Moreover, both the overall trends were strong and significantly decreasing (pre: *C* = .71; *p* < .01; post: *C* = .67; *p* < .01). For the index finger AD, no significant trend was observed during the first day of training either at the pre- or post-TMS phases; similarly, no significant trend was observed the last day of the training. On the contrary, both the overall trends were significantly decreasing (pre: *C* = .68; *p* < .01; post: *C* = .58; *p* < .01). Finally, only the overall trends were significantly decreasing when considering the distance between the wrist and the ring finger (pre: *C* = .82; *p* < .01; post: *C* = .84; *p* < .01). When considering the control participant, data from day 1 were discarded due to a technical problem. Results showed no significant overall trend when the middle finger, the index finger, and the ring finger were moved. Such stable trends were observed in both the pre-TMS and post-TMS phases.

#### 4.1.2. Task 2: Reach-to-Grasp Task

Several variables were considered during task 2. For such variables, the analysis was conducted by referring to the data collected throughout the five days of training before (pre) and after (post) the TMS stimulation. The results obtained from the selected kinematic variables in MD participant are reported as follows.

Movement time: A significantly decreasing trend was observed for the measures collected both pre-rTMS stimulation (*C* = .73; *p* < .01) and post-rTMS stimulation (*C* = .62; *p* < .01).

Maximum grip aperture: A significantly increasing trend was observed for the measures collected before the stimulation (*C* = .64; *p* < .01). No significant trend was observed for the measures collected after the rTMS (*C* = .30; n.s.).

Time of maximum grip aperture: Two clearly and significantly decreasing trends were observed for this variable. The first one involved the measures collected before the rTMS (*C* = .79; *p* < .01), while the second one involved the measures collected after the stimulation (*C* = .62; *p* < .01).

Time to maximum grip velocity: A clearly significant decreasing trend was observed for the data series collected before the TMS (*C* = .72; *p* < .01). The trend observed for the data series collected after the stimulation was significant, although more noisy (*C* = .43; *p* < .01).

Time of maximum wrist height: A significantly decreasing trend was observed at both the pre-rTMS (*C* = .74; *p* < .01) and the post-rTMS (*C* = .69; *p* < .01) phases.

Time of maximum wrist deceleration: With respect to this variable, a significant although very noisy decreasing trend was observed at both the pre-TMS measures (*C* = .49; *p* < .01) and the post-TMS ones (*C* = .50; *p* < .01).

Delay grasping: A significant decreasing trend was observed for this variable for the measures collected both pre-rTMS stimulation (*C* = .57; *p* < .01) and post-rTMS stimulation (*C* = .57; *p* < .01).

**Figure 5:**
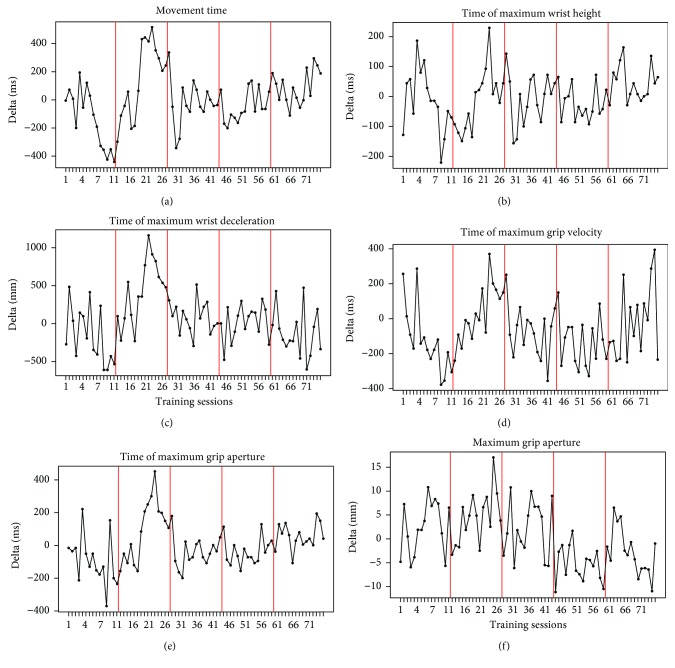
Pointwise delta between the measures, for the main variables of task 2, obtained at pre- and post-rTMS phases by the MD patient. Parameters referring to the reaching component are listed on (a), (b), and (c), whereas parameters for the grasping component are displayed on (d), (e), and (f).

Figure 5 displays the trend of the pointwise delta computed for the main variables measured in task 2. It is noticeable the increase of the negative difference between the pre and the post measures during the first day of training, while an increase in the positive difference between the same values is observed during the second day of training. After these days, the difference tends to remain stable.

**Figure 6:**
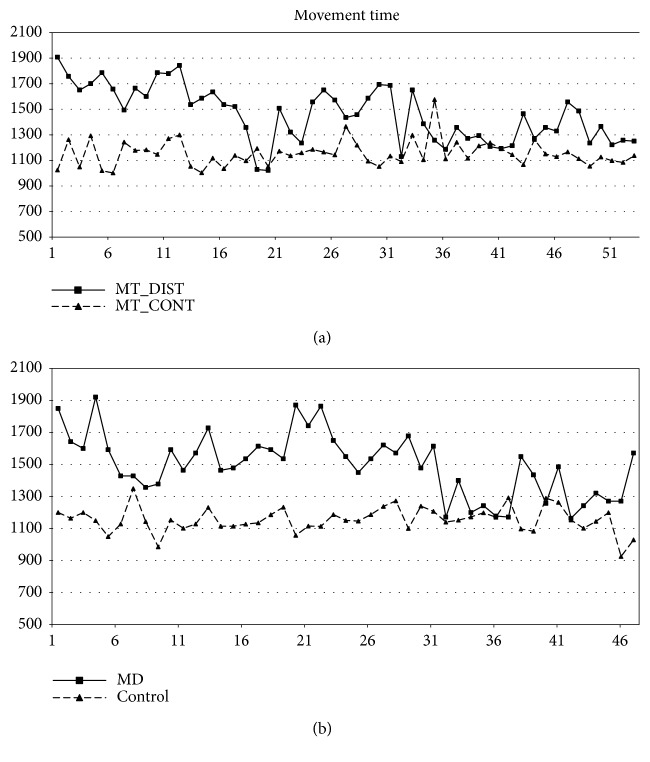
MD and control participant trends on the movement time (task 2).

As concerns the control participant, no trend resulted statistically significant (*p*_s_ > 0.05).  Figure 6 displays together the trends for the MD patient and the control participant. By the figure, it is clearly seen the difference of the two trends in the movement time variable. Similar results were observed for the remaining variables of task 2.

### 4.2. Neurophysiological Measures

No significant trend was observed in motor threshold at rest before (rMT pre) and after (rMT post) rTMS 1 Hz stimulation for either the MD (*C* = .07; n.s.; see Table 1) or the control (*C* = .17; n.s.; see Table 1) participant.

## 5. Conclusions

We set out to investigate neural plasticity in a professional guitarist affected by MD through a multimethodological paradigm. To this end, we combined spTMS, low-frequency rTMS, and 3D motion analysis. Results showed that although rTMS on M1 partially modulated resting motor threshold, a systematic normalization of various kinematic indexes during the 5-day treatment occurred for the MD participant. In particular, as concerns abduction independence, a significant increase in the distance between the dystonia-affected finger and the index and ring fingers was observed on the first day of training during the post-TMS session. The trend significantly increased throughout the five days, showing an overall increase of the distance between the fingers. These data were further confirmed by the distance between the affected finger and the wrist: a decreasing distance (abduction degree) was observed both on the first day of training during the post-TMS session and throughout the treatment's period. These results point to the presence of both a short-term and a long-term trend in the affected finger and not to a general effect of practice.

As regards the reach-to-grasp task, an increase in general motor coordination was hypothesized for the MD participant throughout the five days of training. No significant trend was instead expected for the control participant, since there was no room for improvement (ceiling effect). Results showed an increase in motor coordination only for the MD participant, as indexed by a significant decrease in the movement time. In terms of the reaching component, the time of maximum wrist height and the time of maximum wrist deceleration were anticipated, in line with previous studies demonstrating a significant anticipation when an object is approached more carefully (e.g., [41, 42]). For the grasping component, the amplitude of the maximum grip aperture revealed an increasing pattern of accuracy—as indexed by an appropriate finger scaling—throughout the 5-day training. The time of maximum grip aperture, the time of maximum grip velocity, and the delay grasping were anticipated as well as for the reaching parameters, indicating a temporal coupling between the reaching and the grasping components. These results are consistent with human literature suggesting that task constraints can modulate the proximal and distal components of a coordinated action. The failure to reduce variability as the target is being approached calls for coordination strategies amongst components, which might serve to partially dissipate errors [43].

An intriguing hypothesis points to a malfunctioning in the parietal-premotor pathway of dystonic patients [44]. Parietal-premotor connections are specialized for specific tasks, for example, reach-to-grasp movements, having separate pathways for each of the two components (i.e., reaching and grasping; [45]). Thus, a task-specific deficit could arise from the combination of excessive motor repetition of a particular task, together with disordered control of neural plasticity in the pathway where that specific task was learned [2]. Based on this hypothesis, we might suggest that future behavioral interventions should be based on restoring specific motor pathways through plasticity processes [22].

Notably, an initial positive outcome was observed during the post-TMS session of day 1, when all the parameters jointly showed a significant improvement. This effect, however, was neutralized and reversed during the post-TMS session of day 2, which was then followed by a stabilization phase for the remaining three days. The convergent oscillation of all these parameters seems to indicate that rTMS inhibitory stimulation might be beneficial in the very short term, but it provides a stable advantage only in the course of a 5-day training. This result might suggest that it takes many days of intervention to rebalance motor activity.

Overall, these results suggest that kinematic assessments of abduction independence, abduction degree, and reaching and grasping components are useful parameters for objective quantification of MD before and after training. Moreover, the reach-to-grasp task might allow studying situations similar to those participants facing in their daily life motor activities. This points to the effectiveness of assessing kinematics in conjunction with individual clinical scores such as the Arm Dystonia Disability Scale (ADDS). Although they probe different aspects of motor impairment and might not correlate with each other [46], they should both be used to supplement the clinical diagnosis for monitoring the treatment and to assess the effectiveness of rehabilitation.

In neural terms, no trend was observed when considering rMT. This is a counterintuitive—though not rare—output. Veugen and colleagues [47] recently found that inhibition of the overactive dorsal premotor cortex partially recovered dystonic symptomatology despite having no influence on surround inhibition (i.e., the mechanism in the motor system which focuses neuronal activity to select the execution of the desired movement; [48]), as indexed by MEP sizes. In particular, stimulation improved writing performance in patients, though there was no significant effect on rMT in either dystonic or control participants.

In this respect, the study described here highlights the importance of evaluating brain stimulation outcomes in a more systematic way, beyond classical measures of neural plasticity such as MEP size. The cause of MD is obscure, but a loss of inhibition in the central nervous system and a loss of the normal regulation of plasticity are classically reported [49–51]. Plasticity generally refers to the ability of the nervous system to change the effectiveness of transmission in neural circuits [3]. An increase of sensory and motor finger representations in musicians is usually described as an adaptive plastic change to conform to the new needs. However, when this change develops too far, brain plasticity might shift from a benefit to a maladaptive mechanism [52]. On the basis of this abnormal plasticity hypothesis, new treatment protocols have been designed aimed at the redifferentiation of the disturbed hand representations. Here, we propose a new procedure to investigate affected muscle activations in individuals with neurologic motor disorders after plastic changes induced by rTMS.

This line of intervention holds several advantages over pharmacologic therapy (e.g., injections of botulinum toxin into the intrinsic arm/hand muscles): It is safe and effective, as demonstrated by kinematic analysis, and there is no risk of impairing movement in adjacent fingers. Moreover, it can be applied to patients unresponsive to a variety of commonly used medical treatments. Noninvasive brain stimulation can transiently normalize corticospinal excitability to the affected muscles and can improve the degree of motor coordination over time. Objective quantifications of this treatment can be experimentally obtained with EMG and 3D movement analysis, paving the way for developing novel evaluation tools to optimize therapeutic strategies for motor disorders.

As the rehabilitation research in limb dystonia develops, it will be relevant to investigate comparative effectiveness of interventions to understand which approach holds the most promise.

The present results could support three future research aims:

- To develop an effective diagnostic tool based on neurophysiologic and behavioral measures for early identification of patients and for quantifying changes in symptoms
- To investigate how dystonia affects the parietal-premotor pathway (reaching and grasping components)
- To determine the best frequency and duration for interventions and after effects following rehabilitation.

A limit of the present study is the small sample adopted. However, according to Kimberley and colleagues [23, 53], studies in this field should utilize robust small *n* methodology such as single subject experimental design studies with repeated measures that allows for detailed analysis of within subject variability. Needless to say that definitive statements cannot yet be made regarding efficacy of this paradigm. Randomized controlled measurements are essential for future studies to compare different outcomes with similar frequency and duration.

Identifying the motor dynamics underlying this disorder will be helpful for moving forward both in diagnosis and in treatment, to optimize therapeutic outcomes. Since the available medical approaches are only moderately effective, preventing dystonia is just as much important.

We argue that an enhanced understanding of how neural plasticity can be assessed in FDH affected patients will provide helpful insights for designing more effective patient-tailored therapies based on noninvasive brain stimulation and for evaluating different treatment approaches.

## Figures and Tables

**Table 1:** Resting motor threshold variations throughout the five-day protocol, before (rMT pre) and after (rMT post) the rTMS 1 Hz stimulation at the 90 percent of the rMT.

		Day 1	Day 2	Day 3	Day 4	Day 5
MD	rMT pre	43	43	43	44	44
	rMT post	46	44	46	46	46

Control	rMT pre	43	50	44	52	42
	rMT post	48	60	50	53	56

## Data Availability

All related data are included within the article or will be made available upon request.
